# Epidemiological Alchemy in the AI Era Comes Under Close Scrutiny—But Is It Entirely Without Meaning?

**DOI:** 10.2188/jea.JE20250602

**Published:** 2026-07-05

**Authors:** Kazuki Ide, Takeo Nakayama

**Affiliations:** 1 Division of Scientific Information and Public Policy, Center for Infectious Disease Education and Research (CiDER), The University of Osaka, Osaka, Japan; 2 Research Center on Ethical, Legal and Social Issues, The University of Osaka, Osaka, Japan; 3 Department of Health Informatics, Graduate School of Medicine/School of Public Health, Kyoto University, Kyoto, Japan

Epidemiological open data obtained from the National Health and Nutrition Examination Survey (NHANES), conducted in the United States, have provided valuable opportunities for exploratory research.^1^ What once embodied the public good may now, in the artificial intelligence (AI) era, be revealing the seductive sheen of a “forbidden fruit.”

Suchak et al reported an increase in formulaic research articles using this AI-ready dataset (four articles per year from 2014 to 2021, but 190 in 2024 alone as of October 9), among which a considerable proportion were of questionable quality.^2^ They suggested that this increase was partly driven by AI-assisted workflows and paper mills.

After this problematic trend was reported in *Science*, two major publishers—PLOS and Frontiers—announced that they would no longer consider studies based solely on this dataset without additional research.^3^ This means that authors are now required to include experiments demonstrating robustness or to incorporate additional data from their own institutions to show the unique value of the study. The Editor-in-Chief of PLoS One stated that “the rejection rate for such papers has increased from 40% to 94%,” although it remains difficult to judge whether this change is desirable. A 94% rejection rate seems high. We argue that frequent reuse of a dataset tends to signal Findable, Accessible, Interoperable, Reusable (FAIR)-aligned quality. In this context, Dr Mons, the progenitor of FAIR data principles, has underscored the importance of “Fully AI-Ready” data.^4^ We should remain mindful of the risk of becoming unduly restrictive or overly exclusionary toward exploratory, early-stage work. Not limited to NHANES, the journal *Globalization and Health* has also discussed issues in Global Burden of Disease (GBD) studies, which may stem from comparable problems.^5^

In Japan, collaborative studies, such as the Japan COVID-19 and Society Internet Survey (JACSIS) and the Japan Society and New Tobacco Internet Survey (JASTIS), have conducted multiple waves of data collection.^6^^,^^7^ These groups regularly invite collaborators to use their data, although the datasets are not openly available. Their controlled-access framework represents one possible solution for preventing inappropriate use of data, provided it safeguards a minimum level of quality while remaining open to diverse research. A similar approach is used by the Japan Multi-Institutional Collaborative Cohort (J-MICC) Study, which provides open calls for research proposals to researchers awarded Grants-in-Aid for Scientific Research (KAKENHI).^8^ The initiative is also supported by the Platform of Supporting Cohort Study and Biospecimen Analysis (CoBiA), which includes additional resources.^9^

Another approach is to require authors to post a preprint upon submission, as previously implemented by *eLife*. Such a policy may function as a deterrent against inappropriate use of data and the submission of low-quality manuscripts, since authors’ names remain publicly associated with their work even if it is ultimately rejected. That said, its impact might be limited, given that the generalist server hosted on *OSF Preprints* released a blog post on October 16, 2025 announcing its decision to indefinitely suspend operations, citing concerns over the rising volumes of AI-generated content and paper-mill activities as one reason for the suspension.^10^

Furthermore, discerning whether insufficient quality stems from malicious intent or a mere lack of rigor remains a challenge. Care must be taken not to conflate the level of expertise or methodological rigor with malicious intent, as such assumptions risk stifling emerging research and discouraging ambitious researchers. Importantly, health-related data and the findings derived from them may influence both individual and population-level behavior.

We are living in an age of openness, with this trend being advanced at both national and international levels.^11^ It may be time for us not merely to advance openness, but to proactively rethink its meaning and form—an invitation posed by the very phenomena we are now witnessing.
